# Association Between ABO Blood Group and Gestational Diabetes Mellitus in Pregnant Women at King Abdulaziz University Hospital: A Retrospective Study

**DOI:** 10.7759/cureus.31784

**Published:** 2022-11-22

**Authors:** Nedaa Bahkali, Ghaida Eissa, Hala Sindi, Omar A Almutairi, Kholoud Ghamri

**Affiliations:** 1 Obstetrics and Gynecology, King Abdulaziz University, Jeddah, SAU; 2 Medicine, King Abdulaziz University Faculty of Medicine, Jeddah, SAU; 3 Internal Medicine, King Abdulaziz University, Jeddah, SAU

**Keywords:** saudi women, fetal outcomes, pregnancy, gestational diabetes mellitus, abo blood group

## Abstract

Objective

Gestational diabetes mellitus (GDM) can occur during pregnancy. One of the leading causes of it is a hormone produced by the placenta that interferes with glucose absorption and causes glucose buildup in the bloodstream. Genetic variations between ethnicities are believed to be associated with GDM, and there has been some research on the association of ABO blood group with GDM in different populations. However, the results so far are inconsistent, and there is no conclusive evidence on how ABO blood group affects the occurrence of GDM. This study aims to examine the link between ABO blood group and GDM in pregnant women at King Abdulaziz University Hospital.

Methodology

A retrospective cohort study was conducted on a group of GDM patients between 2019 and 2022 using data collected from the patients’ medical records at King Abdulaziz University Hospital.

Results

The overall prevalence of GDM was high at 74.7%, and the percentage of patients with A, O, B, and AB blood group who had GDM was 42.9%, 41.41%, 12.1%, and 3.59%, respectively. However, there was no significant difference in Rh status or any other clinical characteristic between the participants who had GDM and those who did not have GDM.

Conclusion

The present findings indicate that blood group is not associated with the development of GDM in this cohort from Saudi Arabia. However, more studies are required in the future to corroborate these findings.

## Introduction

In clinical practice, the ABO blood group system is used to classify patients into four blood groups according to the presence or absence of A and B antigens on red blood cells [1]. Antigens for ABO blood groups (A, B, AB, and O) are expressed by a variety of human cells and organs, such as the epithelia, platelets, vascular endothelial cells, and neurons [1,2]. Therefore, the clinical implications of the ABO blood group system outside of transplantation and transfusion treatment is a popular subject of research. In fact, a substantial body of evidence indicates that ABO antigens play a role in a variety of systemic diseases [3-8]. One such disease is gestational diabetes mellitus (GDM), which is defined as glucose intolerance that develops during pregnancy and affects 5% to 10% of pregnancies worldwide [9]. There is some evidence to suggest that different ABO blood groups are associated with a varying risk of developing GDM. For example, a recent study conducted on 792 healthy Iranian women with blood group AB reported that their fasting glucose levels during the second trimester of pregnancy were significantly higher than those of women with other blood types [10]. A retrospective case-control study conducted in Japan also showed that blood group AB was a risk factor for GDM [11]. In contrast, a study conducted on Chinese women revealed that blood group AB provided protection against GDM, and a retrospective cohort study conducted in Israel also found that blood group AB was associated with a lower risk for GDM than the other three blood groups [12,13]. Furthermore, a study conducted in Nigeria showed that Rhesus (Rh) positive blood group, especially O+ blood group, was associated with an increased risk of GDM [14]. Unlike the studies that have shown a link between GDM and ABO blood group, a retrospective cohort study conducted on 5,320 pregnant women in Thailand showed that there was no significant association between ABO blood group and the risk of GDM [15]. Similarly, a retrospective cohort study conducted in Karachi, Pakistan, also found that blood group was not associated with the development of GDM [16]. Furthermore, a 2022 study conducted in Turkey showed that neither blood group nor the presence of Rh factors was associated with the development of GDM [17]. Thus, there are discrepancies in the data which indicate that genetic variation between different ethnicities may affect the association of GDM with ABO blood group type, as demonstrated by a prior study [11]. In Saudi Arabia, the association of GDM with maternal ABO blood group has not been examined recently. Given the differences observed across populations of different ethnicities, it is important to understand whether ABO blood group is associated with GDM specifically in the Saudi population. Therefore, this research aims to investigate the relationship between ABO blood group and GDM in pregnant women who attended King Abdulaziz University Hospital (KAUH).

## Materials and methods

Study design and participants

This retrospective study was conducted on pregnant women who consulted the Obstetrics & Gynecology Department of King Abdulaziz University Hospital, Jeddah, Saudi Arabia, in the western region of the Kingdom of Saudi Arabia (KSA), between 2019 and 2022. The inclusion criteria were pregnant women with no history of type 1 or 2 diabetes and previous history of GDM. None of the diabetic pregnant women was excluded from the study. By taking into account the following statistical assumptions, the sample size was calculated using a single population proportion formula: 95% confidence interval (CI), 50% proportion (because there are no recent studies), 5% margin of error, and 10% non-response rate. In total, 280 participants made up the study's ultimate sample size. Carpenter-Coustan criteria were used to diagnose them with GDM. If a woman had given birth twice or more at the hospital, we utilized the most recent delivery date to minimize any possible bias brought on by including the same woman more than once. The research ethics committee approved this study of King Abdul-Aziz University, Jeddah, Saudi Arabia (Reference No 268-22).

Data collection and analysis

The researchers collected data from the patient's medical records. A pre-designed checklist was prepared for composing data regarding participants' demographic characteristics, including name, date of birth, nationality, blood groups, and Rh status. The other part was about if they have any chronic diseases. The last part was about their obstetric data, including gravidity, parity, and if they had any health problems during the pregnancy. The researchers checked content validity, and the data obtained were entered into Microsoft Excel, 16th edition (Microsoft Corporation, Redmond, WA), and analyzed via IBM SPSS Statistics version 26 (IBM Corp., Armonk, NY). The chi-square test (χ2) was used to determine the significance of differences among qualitative variables according to GDM prevalence. The importance of differences in quantitative non-parametric variables that were expressed as mean and standard deviation (mean ± SD) was examined using the Mann-Whitney U-test. The A p-value of less than 0.05 was considered to indicate statistical significance.

## Results

This study included 280 women who met the inclusion criteria. As shown in Table 1, the mean age of the participants was 37.49 ± 5.37 years, and their mean gravidity was 4.08 ± 2.42. Of the 280 participants, the majority were Saudi nationals (83.9%), and the majority had positive Rh status (91.1%). Furthermore, blood group A was the most common blood group (n = 120, 42.9%). Among the participants who had a past medical history (n = 50, 22.6%), placenta previa was reported in 36%. In addition, hypothyroidism (28%) and hypertension (20%) were the two most common conditions. The mean parity was 3.22 ± 1.94, and parity was 2 in nearly one-fifth of the participants (n = 47, 21.3%). Furthermore, 177 (80.1%) had current pregnancy-related health problems, with GDM (86.6%) being the most common problem. It was followed by gestational hypertension (n = 15, 8.4%).

**Table 1:** Demographic characteristics, blood group, chronic diseases, and obstetric data of the study cohort Note: age and gravidity are presented as mean ± SD UTI, urinary tract infection

Variable	N (%)
Age (mean ± SD)	37.49 ± 5.37
Gravidity (mean ± SD)	4.08 ± 2.42
Nationality	
Saudi	235 (83.9)
Non-Saudi	45 (16.1)
Blood group	
A	120 (42.9)
B	34 (12.1)
AB	11 (3.9)
O	115 (41.1)
Rh status	
Positive	255 (91.1)
Negative	25 (8.9)
Past medical history	
No	171 (77.4)
Yes	50 (22.6)
Past health conditions (n = 50)	
Heart disease	2 (4)
Hypertension	10 (20)
Hyperlipidemia	1 (2)
Hypothyroidism	14 (28)
Obesity	2 (4)
Placenta previa	2 (4)
Other	18 (36)
Parity	
0	7 (3.2)
1	39 (17.6)
2	47 (21.3)
3	39 (17.6)
4	35 (15.8)
5	24 (10.9)
6	16 (7.2)
7	7 (3.2)
8	6 (2.7)
9	1 (0.5)
Mean parity (mean ± SD)	3.22 ± 1.94
Current health problems (related to pregnancy)	
No	44 (19.9)
Yes	177 (80.1)
Pregnancy-related health problems (n = 177)	
Preeclampsia/gestational hypertension	15 (8.4)
Gestational diabetes	165 (86.6)
Thyroid disease	8 (4.5)
UTI	1 (0.5)
Other	8 (4.5)

**Figure 1:**
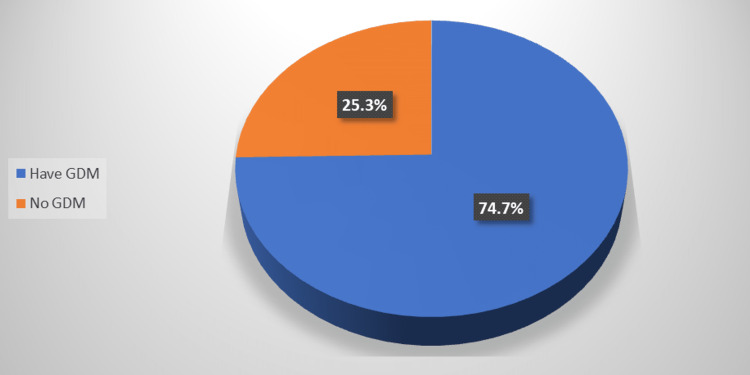
Percentage distribution of the participants according to GDM prevalence GDM, gestational diabetes mellitus

Figure 1 illustrates that 74.7% of the participants had GDM. Table 2 shows that there was no significant correlation between GDM and the demographic characteristics of the participants, blood group, chronic diseases, or obstetric data (p ≥ 0.05).

**Table 2:** Participants’ demographic characteristics, blood group, chronic diseases, and obstetric data according to the presence of GDM Note: all variables are expressed as n (%), expect for age, gravidity, and parity, which are expressed as mean ± SD. GDM, gestational diabetes mellitus

Variable	GDM		χ2	p-value
	Yes, N (%)	No, N (%)		
Age (mean ± SD)	37.93 ± 5.04	36.62 ± 5.64	1.77	0.075
Gravidity (mean ± SD)	4.07 ± 2.45	4.11 ± 2.34	2	0.841
Nationality				
Saudi	140 (84.8)	43 (76.8)	1.9	0.167
Non-Saudi	25 (15.2)	13 (23.2)		
Rh status				
Positive	152 (92.1)	49 (87.5)	1.08	0.298
Negative	13 (7.9)	7 (12.5)		
Past medical history				
No	128 (77.6)	43 (76.8)	0.01	0.903
Yes	37 (22.4)	13 (23.2)		
Parity	3.27 1.93	3.09 1.95		
Mean parity (mean ± SD)			0.75	0.453
Type of current health problems (n = 177)				
Preeclampsia/gestational hypertension	13 (7.9)	2 (3.6)	1.22	0.268
Thyroid disease	6 (3.6)	2 (3.6)	0.001	0.982
UTI	1 (0.6)	0 (0.0)	0.34	0.559

**Figure 2:**
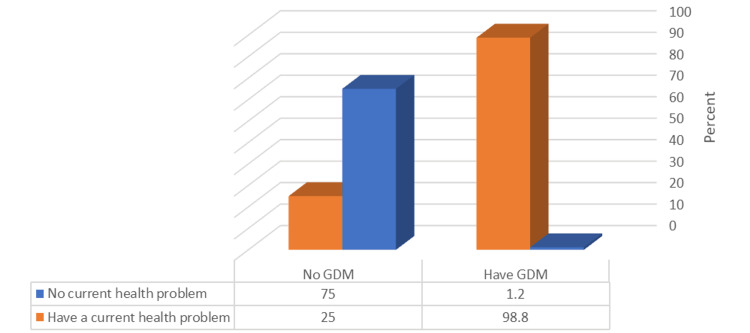
Relationship between GDM prevalence and having a current health problem GDM, gestational diabetes mellitus

**Figure 3:**
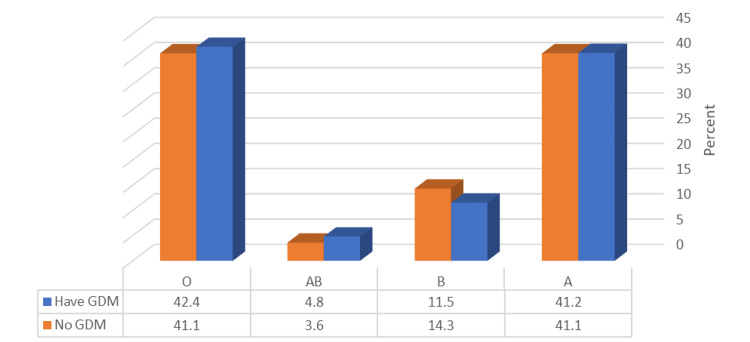
. Relationship between GDM prevalence and blood group type GDM, gestational diabetes mellitus

The proportion of patients experiencing current health problems was significantly higher among those with GDM than among those who did not have GDM (p ≤ 0.05), as shown in Figure 2. However, none of the four ABO blood groups showed a correlation with the occurrence of GDM (p ≥ 0.05), as presented in Figure 3.

## Discussion

This study aimed to assess the association between ABO blood group and GDM in a cohort of pregnant women from Saudi Arabia.

In our cohort, the incidence of GDM was high at 74.7%. This is much higher than the incidence of 24% reported in study conducted in Saudi Arabia on the association of GDM with maternal and fetal outcomes, and it is considerably higher than the incidence of 3.4% reported in a cohort of pregnant women from Pakistan [16,18]. However, in the present study, the mean age of the participants diagnosed with GDM was not significantly different from that of participants who did not have GDM. Similar results were reported in a French cohort, in which the age of pregnant women with and without GDM was found to be similar [19]. However, a case-control study conducted in Thailand found that age was a significant factor associated with the development of GDM, with age above 30 years indicating a significantly higher risk of GDM than age below 30 years [20]. Similarly, a cohort study from Turkey also demonstrated that a significant difference in age between pregnant women with and without GDM [17]. Thus, whether age is a predictor of GDM is still open to debate, and this association must be corroborated through more in-depth analysis that takes confounding factors into account.

Our results showed that the majority of the patients had blood group A, and 91.1% had positive Rh status. Similarly, 46.6% of the pregnant women in a study conducted in Turkey also had blood group A, and a study conducted in France showed that blood groups A and O were the most common and found in 40% and 42% of their participating pregnant women, respectively [17,19]. In contrast, a study conducted in Nigeria reported that 50% of their participants (pregnant women) had blood group O, while a study conducted in Pakistan showed that B+ was the most common blood group [14,16]. This trend reflects the differences in blood group distribution reported in populations of different ethnicities; therefore, it is not surprising that the results differed between most countries that studied the same objectives.

In the current study, no significant relationship was found between GDM prevalence and blood group or Rh status. A study conducted in Saudi Arabia revealed that there is no association between Rh status and type 2 diabetes mellitus in the general population [4]. Therefore, our findings in this cohort of pregnant women may reflect the observation in the general population, but they need to be corroborated through more studies. While our findings reflect the observations made in similar cohorts from Thailand, Pakistan, and Turkey, they contradict the findings reported in the cohorts from France and China [2,15-17]. The previously mentioned retrospective cohort studies from Turkey and France also showed that Rh status was not associated with the prevalence of GDM [17,19]. These findings all reflect differences based on ethnicity, as indicated by a previous study [10]. The present findings also showed that GDM was not significantly associated with other clinical features such as gravidity, parity, past medical history, and current health problems. However, there is not enough information about the association of other demographic and clinical factors with GDM. It might be worth exploring these associations in order to help obstetricians determine the risk of GDM and administer preventive measures in such patients. This might be especially important in the Saudi Arabian population, as GDM was the most commonly reported pregnancy-related health problem in our cohort. Placenta previa was reported in 36% of the study population; this might be because that King Abdulaziz University Hospital is the referral center in western Saudi Arabia for such cases.

One of the main limitations of our study is that our cohort was selected from a single center. Therefore, the findings may not reflect the patterns across the entire Kingdom of Saudi Arabia. Moreover, our sample size was rather small at 280 participants. Larger multi-center studies need to be conducted in the future to corroborate these findings. Another limitation is that we did not obtain data on family history of diabetes mellitus. This could be a predisposing factor that should be explored in future studies. Despite these limitations, our findings lay a sound basis for future studies to explore the patterns of GDM in pregnant women in Saudi Arabia and its association with blood groups, Rh status, and other relevant clinical factors. GDM is significantly associated with composite adverse neonatal outcomes [21]. ABO blood group typing would be an easy method for determining the risk of GDM and initiating antenatal management in the form of insulin therapy or lifestyle modifications to avoid adverse fetal outcomes.

## Conclusions

The present study's findings indicate that neither the ABO blood group nor Rh status is significantly associated with the development of GDM. No recent study examined this association in the Saudi Arabian population; therefore, the findings lay an essential basis for future studies on similar populations from other centers across the Kingdom of Saudi Arabia. Such studies could help determine the risk of GDM based on a simple blood parameter (ABO blood group) and, thus, identify and manage such patients in a timely way and prevent potential adverse fetal outcomes.
